# Graphene Electrode Enabling Electrochromic Approaches for Daylight-Dimming Applications

**DOI:** 10.1038/s41598-018-22274-0

**Published:** 2018-03-02

**Authors:** Joo Yeon Kim, Nam Sung Cho, Seungmin Cho, Kisoo Kim, Sanghoon Cheon, Kyuwon Kim, Seung-Youl Kang, Seong M. Cho, Jeong-Ik Lee, Ji-Young Oh, Yong-Hae Kim, Hojun Ryu, Chi-Sun Hwang, Sujung Kim, Chil Seong Ah, Tae-Youb Kim

**Affiliations:** 1 Reality Display Research Section, Reality Device Research Division, Electronics and Telecommunications Research Institute (ETRI), 34129 Daejeon, Korea; 2 Flexible Information Device Research Section, Reality Device Research Division, Electronics and Telecommunications Research Institute (ETRI), 34129 Daejeon, Korea; 3 Hanwha Techwin R&D Center, Seongnam-si, Gyeonggi-do, 13488 Korea; 4 Electrochemistry Laboratory for Sensors and Energy, Department of Chemistry, Incheon National University, 22012 Incheon, Korea; 5 Reality Device Research Division, Electronics and Telecommunications Research Institute (ETRI), 34129 Daejeon, Korea; 6 Present Address: MCK Tech Co. Ltd., #614, ERICA BI Center Sangnok-gu, Ansan-si, Gyeongi-do, 15588 Korea

## Abstract

For environmental reason, buildings increasingly install smart windows, which can dim incoming daylight based on active electrochromic devices (ECDs). In this work, multi-layered graphene (MLG) was investigated as an ECD window electrode, to minimize carbon dioxide (CO_2_) emissions by decreasing the electricity consumption for building space cooling and heating and as an alternative to the transparent conductor tin-doped indium oxide (ITO) in order to decrease dependence on it. Various MLG electrodes with different numbers of graphene layers were prepared with environmentally friendly poly(3,4-ethylenedioxythiophene):poly(styrene-sulfonate) (PEDOT:PSS) to produce ECD cells. Tests demonstrated the reproducibility and uniformity in optical performance, as well as the flexibility of the ECD fabrication. With the optimized MLG electrode, the ECD cells exhibited a very fast switching response for optical changes from transparent to dark states of a few hundred msec.

## Introduction

To address the increasing concerns of global warming, sustainable and renewable resources must be developed. However in addition, new technologies that can reduce the consumption of the generated energy are also highly necessary, in order to minimize carbon dioxide (CO_2_) emissions^1,2^. Among the global efforts being taken to preserve the earth, the energy losses due to windows in most types of construction, especially in buildings, have to be addressed in order to lower energy consumption, i.e. the energy used for space cooling and heating^3,4^. The functions of windows can generally be divided into technical and esthetic aspects. When the term “control” is added as a technical feature to the window’s typical function, then they are usually called “smart windows”. Smart windows have more active ability to control light and heat transmittance, either manually and/or automatically^4–6^. Among smart window technologies, including photochromic^7^, thermochromic^8–10^ and electrochromic approaches^5,11^, electrochromic devices can provide better performance in terms of ease operation and they are actively switchable to respond to environmental changes as well as personal demand.

The electrochromic (EC) phenomenon is generally defined as the ability to make reversible changes in optical properties, such as transmission, absorption and/or reflection. EC devices (ECDs) typically have structures of containing two conductors sandwiched with electrolytic cells that include an electrolyte and EC materials^12^.

These EC materials have the intrinsic ability to reversibly change their visible color(s) when oxidized or reduced in response to an applied electrical potential, which makes them useful and controllable in EC devices. Among various EC materials, such as organic/inorganic materials, metal-complexes and organic polymers, polymers are the most attractive and promising materials, due to their simple processability, which enables large area fabrication, making them practical and cost effective for smart window applications. Consequently, conducting polymers including polyaniline^13–15^, polythiophene^16,17^ have been widely investigated as potential materials due to their easy processability and effective cost.

In our previous work, one of the well-known conducting polymer materials, poly(3,4-ethylenedioxythio-phene):poly(styrene-sulfonate) (PEDOT:PSS), was investigated in order to optimize EC performance, especially to maximize optical contrast, by adjusting the ion diffusion length, which is affected by PEDOT:PSS layer thickness^12^. The results were promising, in that it was demonstrated that the optical performance of PEDOT:PSS-based ECDs can be significantly improved by structure and/or material development. Besides, environmental-friendly PEDOT:PSS, which is a commercially available product, remains relatively stable under the electrochemical reaction with acceptable reproducibility. Therefore, PEDOT:PSS, with the trade name Clevious P, was also employed in this work. After EC material is selected, the conductors chosen as the electrodes are also very important for electrochemical processes.

Among conductors, tin-doped indium oxide (ITO) is the most commonly used materials in various research and application fields, including photovoltaics^18,19^, light-emitting diodes^20,21^ and also for ECDs^22,23^ because of its outstanding properties such as high conductivity and high optical transparency. However, the ITO surface has to be modified in order to reduce cathodic and anodic corrosion at the ITO surfaces, which affects the durability of the device^24–28^. Moreover, indium is becoming a scarce and expensive resource, which is affecting production schedules and cost. In addition, when poly(3,4-ethylenedioxythiophene): poly(styrene-sulfonate) (PEDOT:PSS) is coated onto an ITO surface, the acidic conditioned PEDOT:PSS leads to an unstable interface between the PEDOT:PSS and indium in the ITO, which then has a tendency to diffuse into neighboring layers, causing the degradation of the device performance and durability over time^27,29^. Consequently, it is mandatory to introduce electrochemically stable electrodes for electrochromic-based smart window and also for chameleon or camouflage applications.

Generally, carbon materials such as carbon nanotubes (CNTs) and graphene are employed as alternatives to ITO for electrochemical devices due to their intrinsic electrochemical stability and good electrical conductivity^14,30,31^. However, compared to the performance of CNTs as electrodes, graphene is a promising material because graphene has lower surface roughness than CNTs, which affects electrical conductivity. Graphene also has outstanding characteristics, including a similar work function level (Φ ~ 4.5 eV) to ITO^32,33^. Although there have been a few reports of graphene assisted ECDs, there has been a lack of systematic investigation, for instance, by varying graphene amount and comparison of graphene flake dispersed solutions and stacked multi-layered graphene with less than 10 layers^34^. Therefore, in this paper the number of graphene layers grown on Cu will be optimized in order to maximize the PEDOT:PSS polymer based ECDs and the electrochemical and electrical characteristics of the different numbers of graphene layers used as electrodes will be investigated.

## Results and Discussions

**Figure 1:**
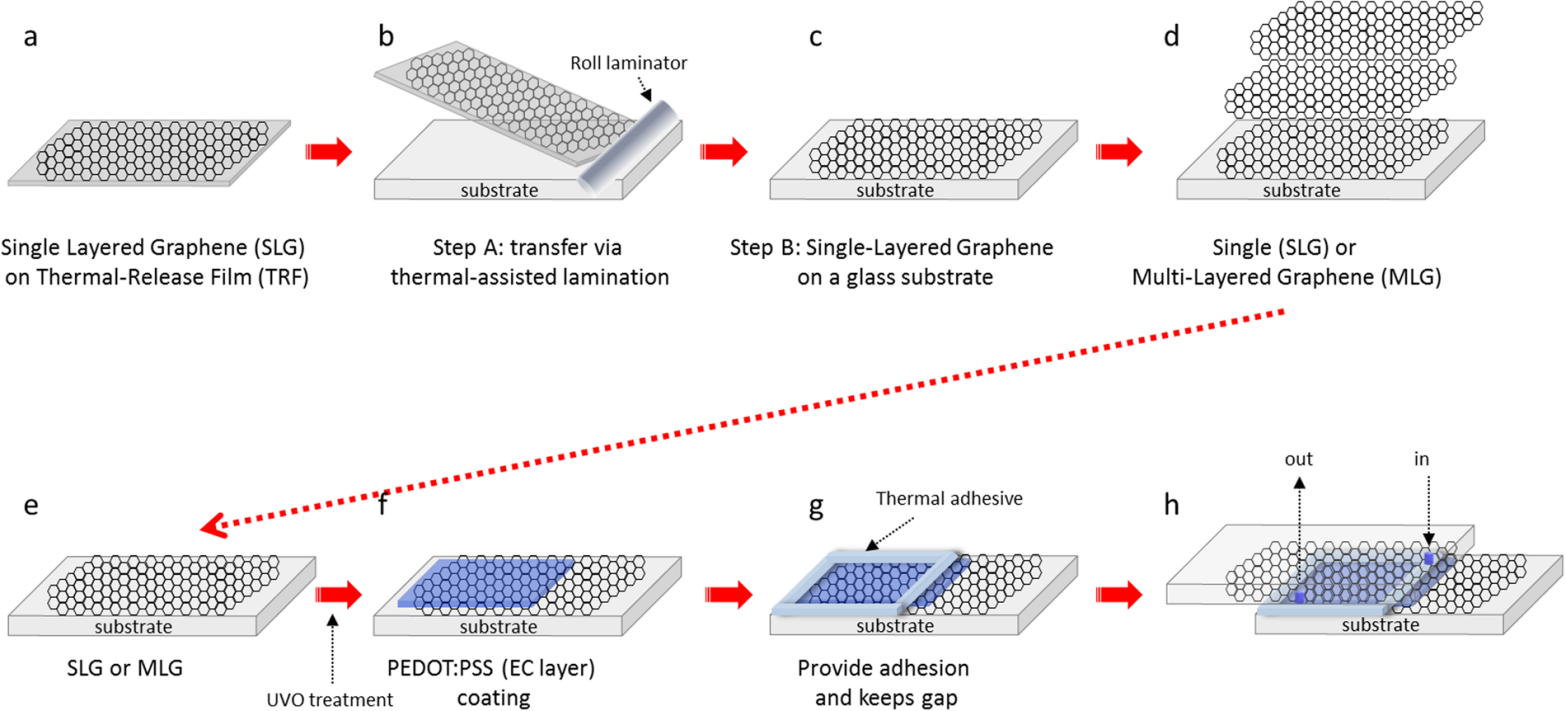
Schematic of the fabrication steps for forming single- and/or multi-layered graphene (SLG and MLG) electrodes and EC cells based on SLG and MLG electrodes. (**a)** A single-layered graphene (SLG) on a thermal-release film (TRF), (**b**) a roll-laminated TRF/SLG with a glass substrate, (**c**) a SLG transferred onto a glass substrate. (**d**) SLG or multi-layered graphene (MLG) produced by repeating the roll-lamination process, (**e**) a UV/ozone (UVO) treatment of the SLG or MLG, (**f**) spin-coated PEDOT:PSS with a certain thickness, (**g**) placement of thermal adhesive to fix the gap and also attach a cover to the SLG and/or MLG electrode and (**h**) A finally prepared EC cell after injecting a liquid electrolyte (L-El) through the in/out holes.

A schematic of the entire process flow for the graphene electrode-based ECD preparation is illustrated in Fig. 1. First, the SLG on TRF (Fig. 1a), which is grown on a copper foil, etched from it and transferred on to TRF, is laminated onto a glass substrate (Fig. 1b) in order to prepare the graphene electrode (Fig. 1c). By repeating step A, a SLG or MLG can be prepared (Fig. 1d). Since graphene has a hydrophobic surface, its surface has to be be hydrophilically modified by UV/ozone treatment in order to obtain a uniform PEDOT:PSS coating, because it is a water-based dispersion (Fig. 1e). After spin-coating the thickness-optimized PEDOT:PSS (Fig. 1f), a thermal adhesive tape with a thickness of 100 μm is placed on the SLG or MLG covered glass substrate (Fig. 1g). Then, an upper SLG or MLG glass substrate with an in/out hole punched in it is applied face-to-face as a cover and it is bonded to the PEDOT:PSS-coated SLG or MLG electrode glass substrate, in order to allow the injection of L-El into the EC cells (Fig. 1h).

**Figure 2:**
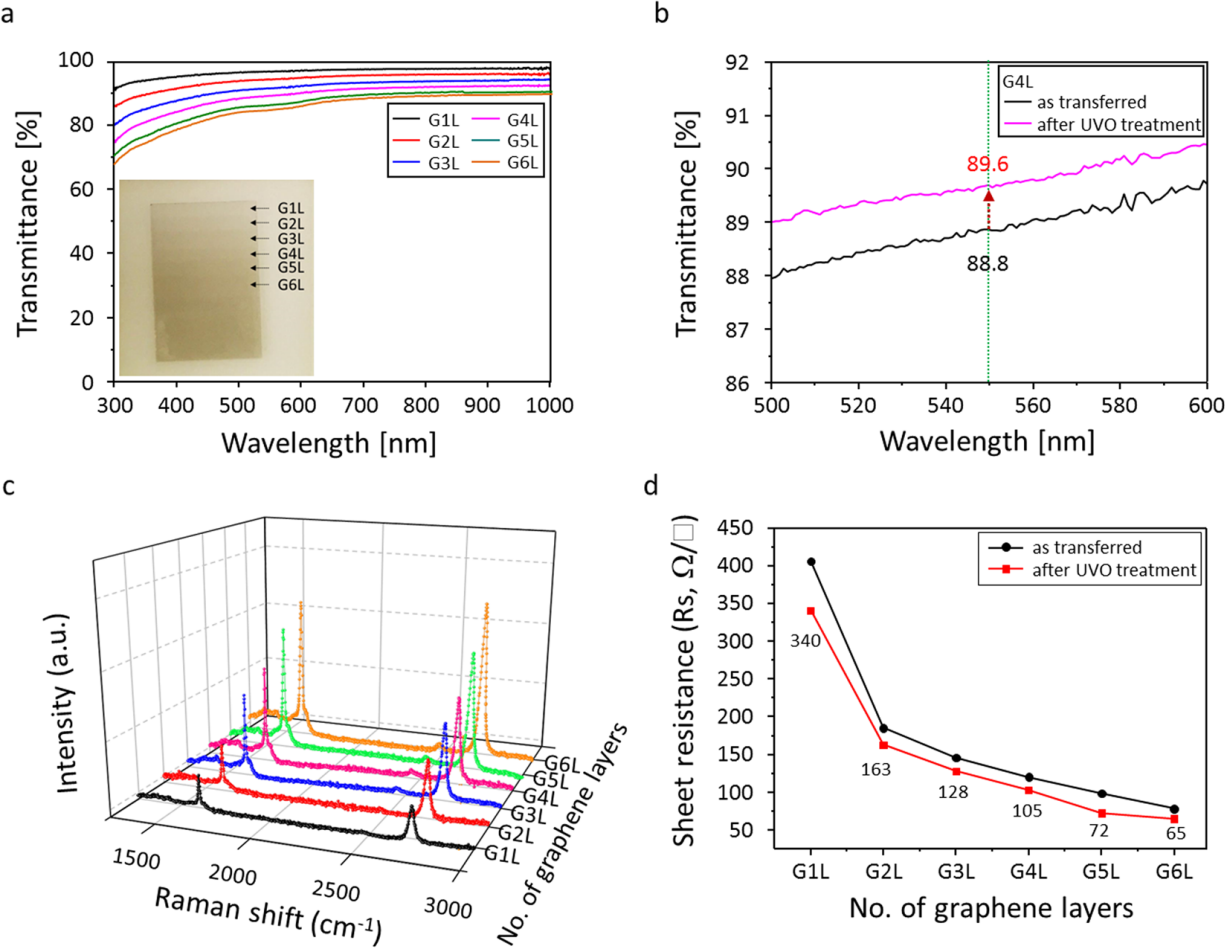
(**a**) Optical transmittance spectra of the SLG and MLG electrodes transferred onto a glass substrate following UV/ozone treatment. The inset provides a photo image of graphene film transferred onto a glass substrate with different numbers of graphene layers, from single (G1L) to multi (G2L, G3L, G4L, G5L and G6L) layers. (**b**) The optical transmittance for G4L increased by at least ~1% after UV/ozone treatment because of the resulting cleaner surface. (**c**) Raman spectra and (**d**) sheet resistance of the graphene films with increasing numbers of transferred graphene layers from G1L to G6L.

In order to investigate ECD performance while using the graphene electrode, the optical and electrical characteristics of the prepared different numbers of graphene layers applied as electrodes was investigated. When the graphene layer was stacked in order to increase the number of graphene layers, the graphene surface was first UV/ozone treated to remove any adhesive residue on top of the transferred graphene surface. As shown in the optical transmittance spectra (Fig. 2a) after UV/ozone treatment, the single layered graphene (G1L) exhibits a high transmittance of ~90% over wavelengths from 300 nm to 1000 nm and over 96% for wavelengths from 500 nm to 1000 nm. The transmittance decreases with the increasing transfer of graphene layers. Although the theoretical loss of transmittance by increasing G1L is 2.3% (@ 550 nm), the experimentally obtained transmittance showed a 2.3~2.8% loss at 550 nm. The inset photo image shows the eye-detectable transmittance of different numbers of stacked graphene layers. The overall optical transmittance from G1L to G6L increased by at least ~1% after UV/ozone treatment (i.e., for G4L, Fig. 2b) because of the resulting cleaner surface.

The different numbers of stacked graphene layers were also confirmed by Raman spectra. In the Raman spectra, there are main peaks which are typically observed as a function of increasing numbers of transferred graphene layers from G1L to G6L. These are the D-peak at ~1300 cm^−1^, the G-peak at ~1600 cm^−1^ and the 2D peak at ~2700 cm^−1^. No peaks in the D-peak position at ~1300 cm^−1^ indicates very high qualified single and stacked graphene layers have been obtained without defects. Moreover, the randomly placed hexagonal lattice among the stacked graphene layers, indicated by the intensity ratio of the G- and 2D-peaks (*I*_G_ and *I*_2D_), was not changed significantly while the *I*_G_ and *I*_2D_ increased together (Fig. 2c)^35,36^.

It is known that the electronic band structure is not strongly affected by randomly stacked graphene layers and the sheet resistance (Rs, Ω/) of the graphene films appears to have decreased with increasing numbers of transferred stacked graphene layers, from G1L to G6L (Fig. 2d). The overall Rs from G1L to G6L after the UV/ozone treatment shows lower values than the Rs of the untreated transferred layers, due to the removal of the adhesive residue. This leads to strong binding between the stacked graphene layers and as a result, Rs dramatically decreased from 340 Ω/ for G1L to 65 Ω/ for G6L which is acceptable for the electrode application.

**Figure 3:**
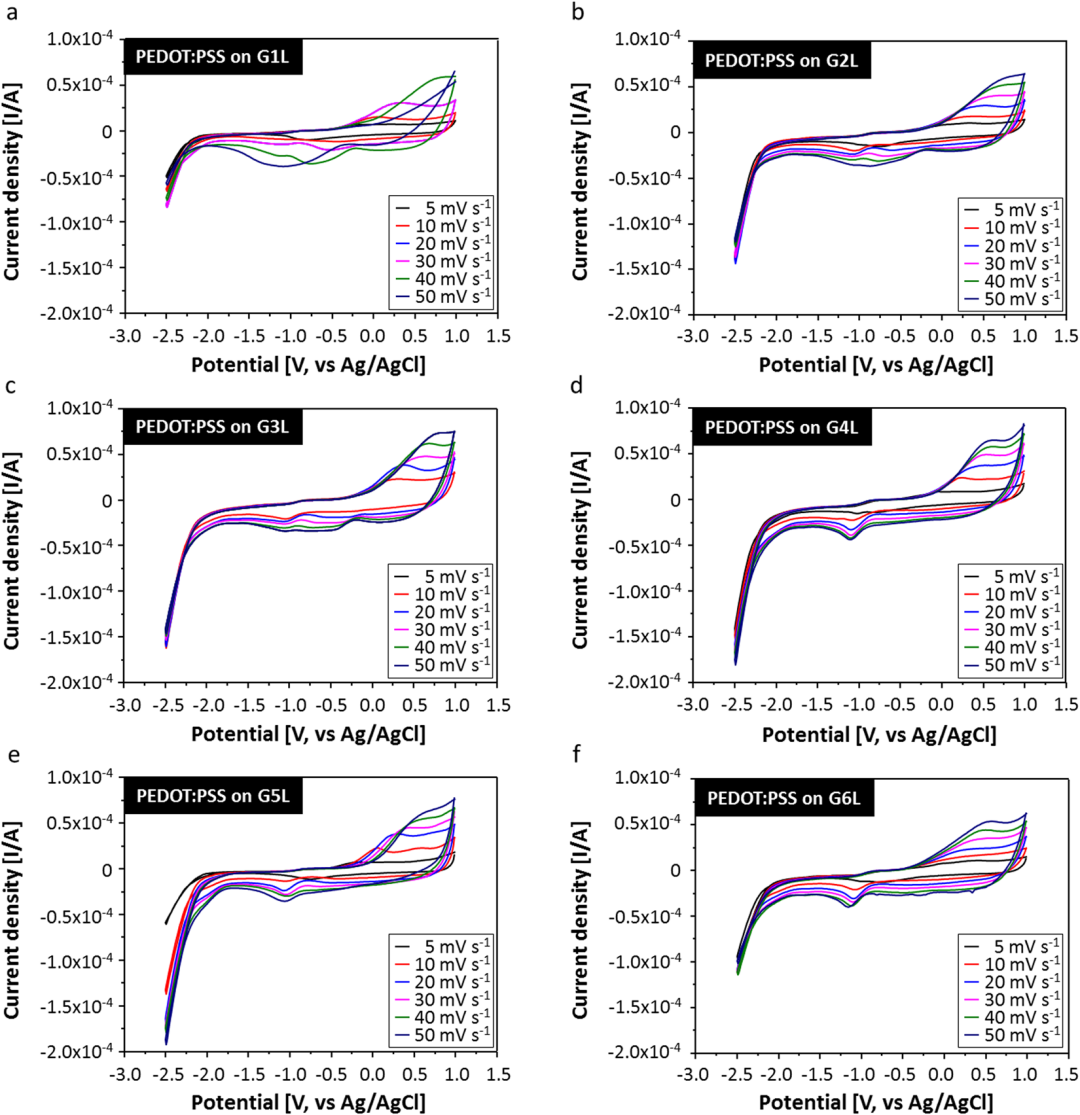
Cyclic voltammograms of PEDOT:PSS spin-coated on different numbers of stacked graphene electrodes from G1L to G6L as a function of various scan rates of 5, 10, 20, 30, 40 and 50 mV s^−1^ in the potential range of −3.0 to 1.5 V. (**a**) glass/G1L/PEDOT:PSS, (**b**) glass/G2L/PEDOT:PSS, (**c**) glass/G3L/PEDOT:PSS, (**d**) glass/G4L/PEDOT:PSS, (**e**) glass/G5L/PEDOT:PSS and (**f**) glass/G6L/PEDOT:PSS.

In order to elucidate the electrochemical response of the graphene electrode based ECD cells, PEDOT:PSS was spin-coated as EC material on the different numbers of the stacked graphene electrodes from G1L to G6L and then reduction-oxidation (redox) behaviors were measured by potentiostatic cyclic voltammetry (CV). The graphene electrodes from G1L to G6L showed redox waves due to the insertion and extraction of lithium ions (Li^+^) between the electrolyte and PEDOT:PSS EC layer with various scan rates of 5, 10, 20, 30, 40 and 50 mV s^−1^ in the potential range of −3.0 to 1.5 V (Fig. 3). As evident from the CVs, all of the graphene electrode based PEDOT:PSS cells showed increased current density with the increases in scan rates, which can be attributed to the diffusion kinetics. Generally, the current density is proportional to the flux which forms near the active surface. Although the magnitude of the current density depends on the scan rates, it is difficult to observe distinguishable clear redox reactions for the PEDOT:PSS EC layers on the G1L, G2L and G3L electrodes, respectively (Fig. 3a,b and c). However, when the PEDOT:PSS EC layers were prepared on the graphene electrodes with increased stacked numbers of graphene layers, such as G4L, G5L and G6L, the redox reaction was found on both the positive and negative sweeps, due to the quasi-reversible processes. Moreover, the oxidation and reduction peaks can be clearly.

observed (Fig. 3d,e and f). Nevertheless, cyclic voltammograms of the PEDOT:PSS EC layer on the G6L electrode showed strongly decreased current density, probably caused by the inefficient insertion and extraction of the Li^+^ ions, which will affect the optical response.

**Figure 4:**
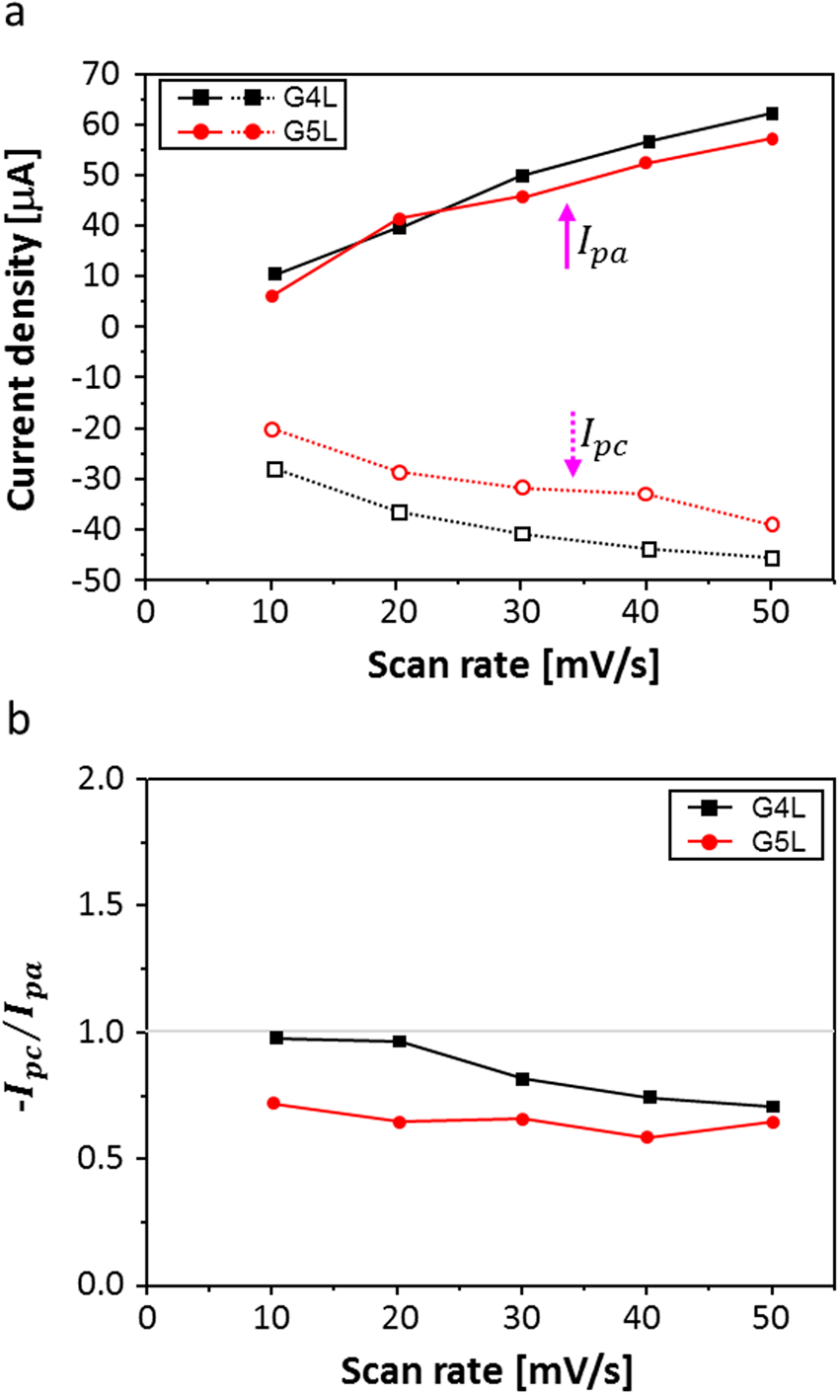
(**a**) The redox peak current densities of the PEDOT:PSS EC layer on G4L and G5L as electrodes for both reduction and oxidation reactions and (**b**) the ratio of anodic and cathodic current density (−Ipc/Ipa) values.

In order to realize the optimum number of graphene layers for the electrode of the PEDOT:PSS-based EC reaction, the redox peak current densities for both G4L and G5L were observed in the CVs (Fig. 3d and e), Both the values for the G4L and G5L appear to be linearly proportional to the increasing scan rates (Fig. 4a). Remarkably, the redox peak current density with PEDOT:PSS on the G4L electrode is significantly higher than the G5L electrode, probably demonstrating efficient interaction with the Li^+^ ions. Additionally, the ratio of anodic and cathodic current density (−*I*_pc_/*I*_pa_) for G4L is close to 1, promising a better reversible electrochemical reaction, while the G5L electrode shows much lower values of −*I*_pc_/*I*_pa_^37^. Therefore, following the comparison of the G4L and G5L electrodes, the G4L was selected and used as the electrode for further ECD performance investigations, due to its enhanced electrochemical activity^38^.

**Figure 5:**
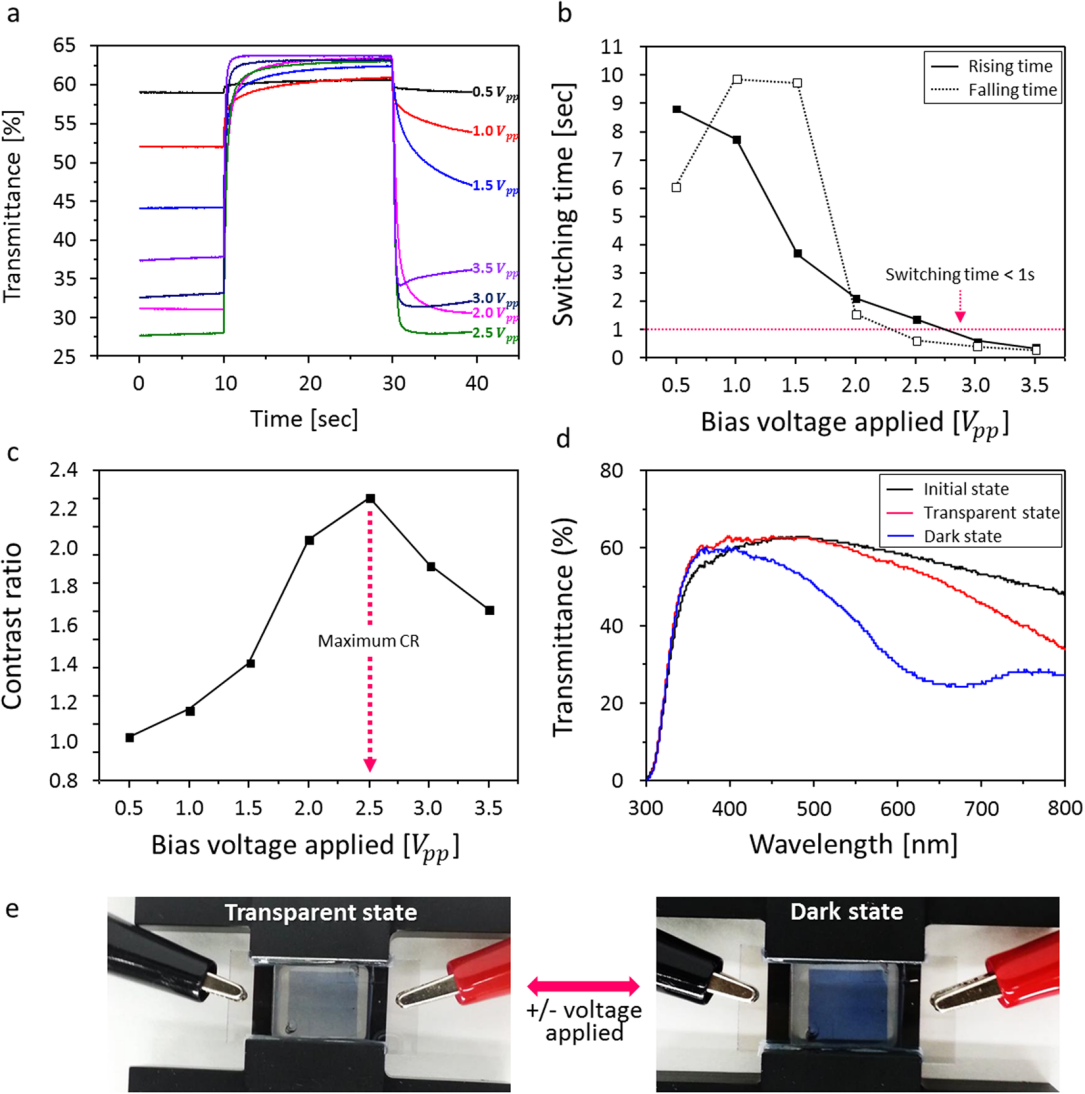
Electrochromic performance of the PEDOT:PSS based ECD cell with G4L as electrode. (**a**) The optical luminous transmittance responses measured as a function of applied +/− voltages ranging from +0.5 to 3.5 V for *T*_*t*_ and −0.5 to −3.5 V for *T*_*d*_ with an offset voltage of −0.5 V during 40 s, (**b**) the plotted optical switching time, generally defined as the time to reach 90% of the optical changes in between *T*_*t*_ and *T*_*d*_, (**c**) the optical contrast ratio calculated from the luminous transmittance difference, also in between *T*_*t*_ and *T*_*d*_, (**d**) the transmittance spectra measured at wavelengths ranging from 300 nm to 800 nm at an applied potential of ±2.5 V for transparent (positive, red line) and for dark (negative, blue line) states and (**e**) the photo images of the optical changes under the applied bias voltages.

In order to investigate the performance of the prepared PEDOT:PSS based ECD cell with G4L as the electrode, the optical transmittance response was measured as a function of the applied positive and negative voltages ranging from +0.5 to 3.5 V for the transparent state (*T*_*t*_) and −0.5 to −3.5 V for the dark state (*T*_*d*_) with an offset voltage of −0.5 V during 40 s (Fig. 5a). The measured luminous transmittance spectra response of the operating ECD cells indicated that there was an optimum voltage to obtain the maximum optical switching time and contrast ratio (CR) value, versus the applied bias voltages. The optical switching time is generally defined as the time needed to reach 90% of the optical changes between the transparent and dark states (Fig. 5b). Over the applied potential from ±2.5 to ±3.5 V, the switching time from the dark to transparent state showed a fast response time, shorter than 1 second and it showed much faster response time of approximately 500 msec from the transparent to dark state, which is noteworthy. This performance confirms that using G4L as the graphene electrode significantly improves the ECD performance, even with PEDOT:PSS, which typically exhibits a very slow switching time of over 5 sec. Moreover, the optical CR can also be calculated from the difference in luminous transmittance between the transparent and dark state and the value showed a drop after the potential of ±2.5 V was applied (Fig. 5c). This is because the optical changes were not efficiently switched from the transparent state to the dark state when reduced, whereas the transparent states were similar to each other. This probably occurs when the PEDOT:PSS reaches an over-oxidized and/or irreversible state due to the application of excessive bias voltage, or the bias voltage is applied for a long time. This behavior indicates that the ECD performance is voltage-dependent and that excellent optical contrast can be obtained when the optimum voltage is applied. Consequently, the transmittance spectra of the PEDOT:PSS based ECD cells with G4L electrode was measured at wavelengths ranging from 300 nm to 800 nm at an applied voltage of +2.5 V for transparent (red line) and −2.5 V for dark (blue line) states, corresponding to the photo images, which show clear optical changes (Fig. 5d and e).

## Conclusions

Among the multi-layered graphene (MLG) electrode-based ECD cells prepared with PEDOT:PSS, the 4-layered graphene (G4L) electrode showed the best electrochemical behaviors in terms of electrochemical stability and reactivity, which enables the replacement of ITO as the electrode. Moreover, the optical switching time, which is one of the important parameters of ECD performance, was significantly enhanced and showed a fast optical change response of less than 1 sec from the dark to transparent state and only 500 msec from the transparent to dart states. Furthermore, the maximum contrast ratio was reached at ±2.5 V, which is low enough to improve device stability. Although there are still remaining issues, such as the mass production and reproducibility of graphene electrodes, this work can provide positive insights for the realization of graphene based devices employing redox-based processes.

## Materials and Methods

### Materials

Single-layered graphene on thermal release film (TRF) was provided by Hanwha Techwin Co. Kr. (Korea). PEDOT:PSS was purchased from Heraeus Precious Metals GmbH & Co. (Germany) and used as received. Also, 1,2-dichlorobenzene (DCB, 99.0%), propylene carbonate (PC, 99.7%, anhydrous), tetrabutylammonium hexafluorophosphate (TBAPF_6_, 99.0%) and lithium trifluoromethane-sulfonate (LiCF_3_SO_3_, 99.9%) were acquired from Sigma-Aldrich (USA) and used without further purification.

### Multi-layered graphene electrodes preparation

A graphene layer with polymer supports, grown on a copper foil, is released by etching the copper foil using aq. 0.1 M ammonium persulphate solution (NH_4_)_2_S_2_O_8_ and then the released graphene layers are transferred onto the TRF using the method described in the literature^29,39^. Finally, the single-layered graphene (SLG, referred to here as G1L) is detached from the TRF and laminated to form multi-layered graphene (MLG referred to as G2L, G3L, G4L, G5L and G6L). MLGs are prepared by stacking SLG on the desired substrates using the lamination technique at 160 °C^40^.

### Liquid electrolyte (L-El) and electrochromic device (ECD) preparation

As the electrochromic material, the thickness-optimized PEDOT:PSS was spin-coated on the prepared SLG or MLG, then PEDOT:PSS coated SLG or MLG was covered with the same-layered graphene with a 100 μm-gap fixed by thermal adhesive tape to prevent infiltration by oxygen and moisture. Then, a mixture of LiCF_3_SO_3_ (10 mM) and TBAPF_6_ (100 mM) dissolved in a co-solvent system of DCB and PC (v:v = 3:1) was prepared as the liquid electrolyte (L-El) and injected into the ECD cells with the final structure of SLG or MLG/PEDOT:PSS//L-El//SLG or MLG. The active area was defined by the SLG or MLG area which was 1.0 × 1.0 cm.

### Characteristics

The film thickness of the spin-coated PEDOT:PSS was measured using an Alpha-step 500 profilometer. The optical transmittance measurement of the laminated graphene layers was performed using UV-visible-NIR spectroscopy (Perkin Elmer Lambda 750 Spectrometer, USA). Raman spectra of the laminated graphene layers were obtained by Raman spectroscopy (Horiba high resolution dispersive Raman microscope, France). The beam size of the microscope equipped with a 532-nm laser and a 50X objective was just 1 μm and the applied maximum power was 10 mW. Electrochemistry measurements were performed with a potentiostat (CHI1030, CH Instrument Inc., USA). Cyclic voltammetry was conducted with a three-electrode cell in which ITO (with an active area for both the ITO and PEDOT:PSS of about 1 × 1 cm) was used as a working electrode. A platinum wire and saturated Ag/AgCl (SCE) were used as an auxiliary electrode and a reference electrode, respectively. The optical contrast ratio and response times were measured via the converted luminous transmittance (resulting in absorption and transmission changes) after applying a reverse bias voltage ranging from −3.5 to +3.5 V for both bleached and colored states, respectively, using an LCD electro-optical measurement system (LCD 5200, Otsuka Electronics Co., Ltd., Japan) in transmission mode with a standard illuminant D65 (halogen lamp) as a light source.
